# The Quality and Reliability of Information in the Summaries of Product Characteristics

**DOI:** 10.3390/ijerph19042185

**Published:** 2022-02-15

**Authors:** Ewelina Drelich, Urszula Religioni, Kevin Chung, Justyna Kaźmierczak, Eliza Blicharska, Agnieszka Neumann-Podczaska, Jerzy Krysiński, Piotr Merks

**Affiliations:** 1 Department of Pharmaceutical Technology, Faculty of Pharmacy, Collegium Medicum in Bydgoszcz, 85-067 Bydgoszcz, Poland; ewelina.drelich@pgf.com.pl (E.D.); jerzy.krysinski@cm.umk.pl (J.K.); 2 School of Public Health, Centre of Postgraduate Medical Education of Warsaw, 01-826 Warsaw, Poland; 3 Children’s Hospital of Eastern Ontario, Ottawa, ON K1H 8L1, Canada; kchung@cheo.on.ca; 4 Zdrowit sp. z o.o., Pharmacy Chain, ul. Diamentowa 3, 41-940 Piekary Śląskie, Poland; j.kazmierczak@aptekizdrowit.pl; 5 Department of Analytical Chemistry, Medical University of Lublin, Chodźki 4a, 20-093 Lublin, Poland; elizablicharska@umlub.pl; 6 Chair and Department of Palliative Medicine, Poznan University of Medical Sciences, 61-245 Poznan, Poland; ar-n@wp.pl; 7 Department of Pharmacology and Clinical Pharmacology, Faculty of Medicine, Collegium Medicum, Cardinal Stefan Wyszyński University in Warsaw, 01-938 Warsaw, Poland; piotrmerks@googlemail.com

**Keywords:** SmPC, medicinal product, patient safety, medication handling

## Abstract

The Summary of Product Characteristics (SmPC) is an obligatory document concerning a medicine required (among other things) for the authorization of a medicinal product. The purpose of the SmPC is to provide product information to healthcare professionals. A necessary condition for this is to ensure that the SmPC is clear and precise. However, neither European nor national legislation obliges marketing authorization holders to review the SmPC in terms of its readability and understandability prior to the registration of a medicine. To date, research on SmPCs has focused on accuracy and completeness; however, the literature lacks information on the extent to which SmPCs meet the needs of healthcare professionals concerning the readability of the information they contain. The main objective of this article is to point out the lack of precision in the legal provisions for the preparation of SmPCs concerning the comprehensibility of the provisions. The article points to the lack of testing of the SmPC in terms of accessibility and transparency for healthcare professionals, highlighting that the document does not meet the needs of healthcare professionals in providing adequate information about medicines. It shows that the current rules and guidelines for the preparation of the registration dossier for a medicinal product are not entirely precise and contain numerous shortcomings.

## 1. Introduction

The Summary of Product Characteristics (SmPC) is a legal document, is one of the obligatory elements of the registration dossier of a medication, and is necessary to issue a marketing authorization for a medicinal product. The obligation to draw up a Summary of Product Characteristics is laid down in several European Parliament laws. The first documents were Directive 2001/83/EC of the European Parliament and the Council relating to medicinal products for human use, and Regulation 726/2004. The document was amended several times and finally replaced in 2004 by Directive 2004/27/EC. The latest consolidated version of Regulation 726/2004 is dated 5 June 2013 [1,2].

The Summary of Product Characteristics (SmPC) is the main document containing details of a particular medicine. The document submitted by the marketing authorization holder shall contain key information for healthcare professionals on the safety and efficacy of the medicinal product. A Guideline on Summary of Product Characteristics issued in September 2009 by the European Commission provides detailed guidance on what should be included in specific sections [3]. Despite the structured form of this document, healthcare professionals (doctors, nurses, and pharmacists) repeatedly stressed that the development of the document requires a fresh approach, with the use of other provisions that are more comprehensible to professionals and whose interpretations will not be divergent or questionable. This problem was particularly highlighted when biosimilar medicinal products began to appear in large numbers on the pharmaceutical market.

SmPCs should be regularly reviewed and updated as new information becomes available, as misleading information in SmPCs may result in adverse effects, unnecessary treatment, or treatment failure. It is recognized that the SmPC is the most commonly used reference document for doctors even though there are other important documents published in the EU that contain product information. The European Public Assessment Report (EPAR) summarizes the review and conclusions of the scientific evaluation by the Committee for Medicinal Products for Human Use (CHMP). EPAR reports are primarily intended to provide information on how the medication was assessed by the CHMP and describe the scientific conclusions of the relevant Agency committee. Physicians probably do not realize that the EPAR document itself is not updated [4], but rather is supplemented by additional documents such as summaries called ‘Procedural steps taken and scientific information subsequent to authorization’.

This paper aims to identify gaps in the legislation and guidelines for the preparation of a medicinal product registration dossier. The article was compiled from information in Medline and PubMed databases. The following search strategies were used: ‘Summary of Product Characteristics (SmPC)’; ‘smpc’, ‘smpc differences’, ‘drug information’; ‘readability’; ‘user testing’. Papers written after 2000 were taken into account. When selecting the appropriate publications, cross-sectional studies and overviews in various groups of drugs and various groups on both original and generic products, regardless of the therapeutic group of medical professions, were taken into account. Publications describing individual safety data sheets and individual cases of gaps in the documentation related to a specific drug were omitted.

## 2. Results

### 2.1. Understanding of the Information Contained in the SmPC

One of the primary roles of the SmPC is to provide information to healthcare professionals. For this to be achieved, it is necessary to meet the basic conditions for effective data communication (the understanding of how different recipients perceive the message, as well as the key factors in changing patient and clinician behavior). The European Commission has included in its guides a laconic statement that SmPCs should be written in clear and concise language [3]. Furthermore, the legislation and the EU guidelines that are based on it only set standards for the readability of a package leaflet addressed to patients, but not the SmPC dedicated to healthcare professionals. Table 1 details the advantages of well-prepared medical records and the disadvantages of poor-quality records.

The EU directives, Polish pharmaceutical law, and other regulations do not oblige marketing authorization holders to test the SmPC for readability and comprehensibility before registration. To date, research on SmPCs has focused on the accuracy and completeness of content [6,7,8,9,10]. However, very little is known about how SmPCs are used by healthcare professionals, or if they meet the needs of health professionals as a trusted source of information.

One study found that SmPCs in their current format do not meet the requirements of doctors in terms of providing sufficient information [11]. This conclusion was based on poor performance in finding and understanding specific information. In addition, it has been shown that a small number of primary care physicians and family doctors use this document as a source of information on the medicine [11]. Based on this study, the following recommendations were made for changes to the structure of the SmPC:

1. Improving and simplifying the structure of headings, in particular replacing ‘Clinical data’ with ‘Dosage and use’.
2. Increasing the visibility of headings and sub-headings.
3. Adding a key information section at the beginning of the document.
4. Adding a list of main headings after the key information section.
5. Using simple language and shorter sentences.
6. Using bullets to improve readability.
7. Using a direct writing style; active rather than passive.
8. Putting related information in one place.
9. Putting information in places where readers can find it.
10. Making the SmPCs available in both paper and online versions [12].

### 2.2. Differences in the Content of SmPCs for Medicinal Products with the Same Active Substances

Another problem with SmPCs is discrepancies in the registration dossiers of preparations with the same qualitative and quantitative composition of active substances. One of the analyses carried out covered 31 medicinal products and showed that more than 60% of them contained significant (critical) differences between original and generic products [12]. Fourteen of the thirty-one selected medicines had discrepancies in the content of the contraindication section. Of these, 71.5% of the medicines were assessed as demonstrating critical differences and 28.5% as demonstrating very minor differences in the content of this section. Moreover, registration dossiers differed from country to country [13], probably due to different legislation. An assessment of the consistency in the information contained in the SmPCs of generic antimicrobial medicines led to similar conclusions [14]. The omission of clinically relevant information related to pharmacokinetic properties was noted.

Disparities in the SmPCs of generic and original medicines are unfortunately a reality. According to the EMEA guidelines, all relevant aspects of the content of generic SmPCs should be consistent with the SmPCs of the reference medicinal products—the so-called ‘branded’ medicines [15]. This is because inconsistent information on medicines containing the same active substance contributes to confusion and poor prescribing decisions. Although similar regulatory requirements apply to generic medicines in the US, inconsistency has been noted among bioequivalent medicines, both there and in other countries for the same medicine authorized by the same regulatory agency. These discrepancies are particularly striking as they put specific subpopulations such as young children or patients with comorbidities at risk. Discrepancies in the SmPC could potentially be life-threatening or even fatal to the patient, which raises serious concerns. One such possibly deadly discrepancy concerned adrenaline: one generic product’s SmPC lacked contraindications for patients with ventricular fibrillation, cardiac dilatation, or coronary insufficiency. This discrepancy may lead to ventricular arrhythmias and/or coronary ischemia in such patients. The second potentially fatal discrepancy concerned promethazine: generic product labels lacked a contraindication for patients up to 2 years of age, leading to a risk of respiratory depression in this subpopulation. These discrepancies represent extremely important omissions from patient safety labels [12,16].

This suggests that if responsible parties do not continuously compare labels with their competitors (generic or originator companies), publicly available scientific information will not be updated to include new side effects. Moreover, this key information will not be available to patients and doctors. This fact may lead to a lack of awareness among patients and physicians about potentially important safety information for a specific medicinal substance.

This fact may result in inconsistent information being communicated to healthcare professionals and patients regarding the same active ingredient. It also raises questions about the duty to provide information and liability for medical malpractice. This is also problematic from the point of view of the pharmacist, who has the right to issue a substitute to the patient. Under the current legislation, a ‘medicinal product having the same qualitative and quantitative composition in active substances, the same pharmaceutical form as the reference medicinal product and whose bioequivalence with the reference medicinal product has been demonstrated by appropriate bioavailability studies’ [17], is considered a substitute. Pharmaceutical law does not mention differences in the registration dossier for a medicinal product. The problem also relates to other aspects of the safety of medicinal products, such as side effects and risk assessment by the doctor.

### 2.3. Incomplete Information in SmPCs

Despite guidelines on the drafting of SmPCs, there are still some in widespread circulation that contain gaps in specific sections, e.g., information on dosages in patient subpopulations. Knowledge of dose adjustments and contraindications for drugs in patients with, for example, hepatic impairment, is often based on the results of endpoint studies conducted by pharmaceutical companies. In recognizing that information was not always generated to the same extent for different drugs, the European Medicines Agency (EMEA) published a guideline on the assessment of endpoints in patients with hepatic impairment in 2005 (European Medicines Agency, 2005). Those guidelines provide recommendations for the design and reporting of test results in patients with hepatic impairment. The results of those studies needed to be included in the study report and discussed in the EPAR, and then included in the SmPC. However, of the 51 medicinal products registered in 2015- 2017, as many as 15 did not have the required dosage information for patients with hepatic impairment [18]. On average, 7 out of 9 pieces of information requested by the EMEA were available in these SmPCs. Safety information or dosage recommendations for patients with severe hepatic impairment were unavailable for almost 60% of the drugs evaluated and/or were ambiguously worded. Basic information on the type of liver disease of the patient included in the required studies was missing for 35 of the 36 drugs. For 21 drugs, this information could be found in the unpublished part of the study report [18]. Vague statements such as ‘not recommended’ or ‘use with caution’ leave open the interpretation of whether the drug is absolutely contraindicated and what the possible effects of its use would be. Ambiguous wording was also observed in studies analyzing SmPC recommendations in other clinical areas, such as renal impairment [18].

Similar issues concern the section related to the use of drugs in pregnant and lactating women covering: drug concentrations crossing the placenta, secretion of the drug into milk, the existence of preclinical and clinical studies, and clinical experience describing the use of the drug during pregnancy and lactation, the effects of the drug on human fertility, the use of drugs in women of childbearing age and specific recommendations for use during pregnancy and breastfeeding. In one study, out of 534 SmPCs, 89.3% did not mention whether the drug crosses the placenta; 67.6% indicated that there was no clinical experience during pregnancy, and in 61.4%, it was not known whether the drug is secreted into human milk. Recommendations for use during pregnancy and breastfeeding were ambiguous in 57.0% and 16.5% of the SmPCs, respectively, and drug use was restricted in more than 90% of the SmPCs for both pregnancy and breastfeeding, despite a lack of information to support these restrictions. Furthermore, the time that has elapsed since the first approval of the SmPCs has not been associated with an increase in the quality of information [19].

## 3. Conclusions

The above summary does not shed light on all the problems associated with SmPCs. Nevertheless, it is possible to conclude from this that the SmPCs issued by pharmaceutical companies and ultimately approved by regulatory agencies are becoming an ‘official’ source of information about medicines that cannot be fully relied upon by the medical profession. Potential prescribing and monitoring errors were estimated to occur for 5% of items prescribed in general practice. Mistakes made by doctors in training in hospitals are most often due to a lack of knowledge and the inappropriate presentation of drug information [20]. It is therefore important that prescribers have ongoing access to high-quality, evidence-based drug information that is timely, accurate, and practical to ensure safe and effective prescribing. The agencies involved in registering medicines in the various EU countries should consider clarifying the requirements for drawing up registration documents for medicines. Consideration should be given to requiring detailed information on the use of medicinal substances in pregnant and breastfeeding women when submitting applications for authorization. To date, no studies have been conducted in Poland to analyze the information contained in the SmPCs of medicinal products.

## Figures and Tables

**Table 1:** Advantages and disadvantages of good and poor medical records [5].

Well-Prepared Medical Records	Poorly Prepared Medical Records
- Support the exchange of relevant information and strengthen multidisciplinary team communication - Assist in the coordination and continuity of patient care - Help patients to make informed decisions about their treatment - Improve the availability of risk assessment data - Improve the availability of data for analysis in case of treatment failure - Help guide diagnostics and treatment plans	- Mislead medical staff and patients - Increase the occurrence of a medico-legal error - Unnecessary repetition of diagnostic and laboratory tests - Extend hospitalization - The risk of incorrect treatment of the patient - Occurrence of serious undesirable effects - Increase in patient mortality

## Data Availability

All data are available from the corresponding author.
